# Resolving the Challenges of an Enormous Popliteal Artery Aneurysm Rupture

**DOI:** 10.7759/cureus.77862

**Published:** 2025-01-23

**Authors:** Christiana Anastasiadou, Antonia A Bourtzinakou, Christos Giankoulof, Konstantinos Siozos, Stamatis Angelopoulos, Angelos Megalopoulos

**Affiliations:** 1 Vascular Surgery Department, General Hospital of Thessaloniki "George Papanikolaou", Thessaloniki, GRC; 2 Fourth Academic Surgical Department of Aristotle University of Thessaloniki, General Hospital of Thessaloniki "George Papanikolaou", Thessaloniki, GRC; 3 Radiology Department, General Hospital of Thessaloniki "George Papanikolaou", Thessaloniki, GRC

**Keywords:** endovascular, giant aneyrysm, open, popliteal artery aneurysm, rupture

## Abstract

Popliteal artery aneurysm rupture represents a diagnostic and therapeutic challenge. In this case report, we report the open surgical therapy of an enormous ruptured popliteal artery aneurysm (rPAA) (13 cm), emphasizing key points in diagnosis and treatment decision-making. The symptomatology of rPAAs is often misleading, with nonspecific signs such as edema and pain, which can lead to delayed diagnosis. Early recognition and appropriate treatment are critical to avoid complications such as acute limb ischemia and amputation.

## Introduction

Popliteal artery aneurysm (PAA) is an abnormal expansion of the artery located behind the knee (>1.5-2 cm), and it is the most common type of peripheral artery aneurysm, accounting for approximately 70% of all peripheral aneurysms [1]. PAAs usually remain asymptomatic or present with acute limb ischemia due to thrombosis or distal embolization. Rupture is a rare clinical finding accounting for approximately ~2% of cases [2]. The differential diagnosis and management of these cases may be challenging due to several factors: the masquerading nature of the disease, the need for urgent diagnosis, and the risk of damage to adjacent structures like nerves. The aim of this study is to present a case report, highlighting key points in diagnosis and therapy decision-making.

## Case presentation

**Figure 1:**
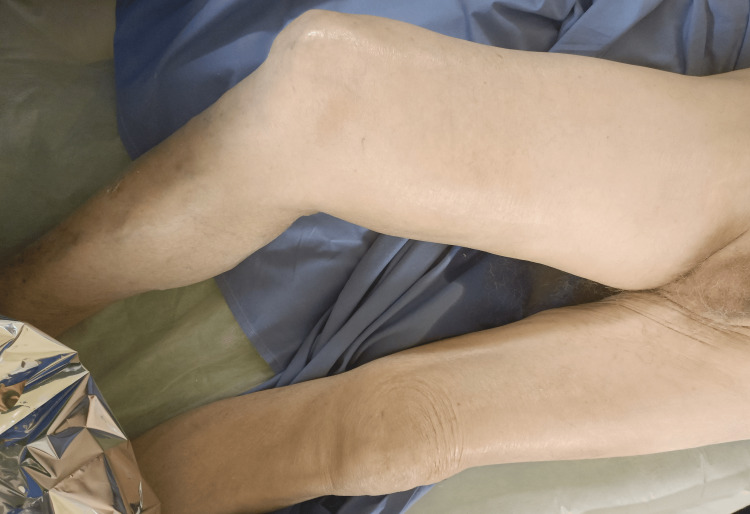
Clinical presentation included an extremity with excessive edema

An 87-year-old man presented to the emergency department complaining of pain and edema of his right lower extremity, which progressively worsened over the past week (Figure 1).

On physical examination, there was noted pulselessness of his right lower extremity accompanied by the presence of an enormous mass in the popliteal fossa. Also, there was noted mild paresthesia and drop foot, which are signs both of acute limb ischemia and of peroneal nerve compression from the gigantic aneurysm. Laboratory examination revealed a critically low value of hemoglobin (5.9 mg/dl) (Table 1). D-dimer testing could be elevated in these cases, but one should keep in mind that it lacks specificity, and it can be elevated in a wide range of diagnoses, such as acute limb ischemia, dissection, aneurysm rupture, and deep vein thrombosis.

**Table 1:** Blood test results at the time of the presentation CPK: creatine phosphokinase; CRP: C-reactive protein; WBC: white blood cell; Hb: hemoglobin; Ht: hematocrit

Lab test	Value	Unit	Normal range
Urea	90	Mg/dl	10-55
Creatinine	1.08	Mg/dl	0.7-1.1
CPK	45	U/L	20-180
CRP	13.05	Mg/dl	<0.8
WBC	13.57	K/μL	3.8-10.5
Hb	5.9	g/dl	14-18
Ht	18	%	40-52
Troponine	15	Pg/ml	<58

**Figure 2:**
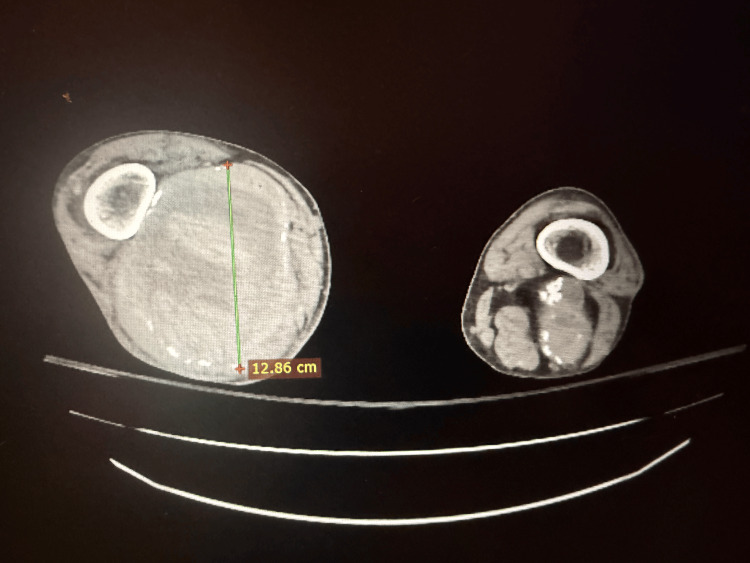
CT angiography revealing an enormous PAA PAA: popliteal artery aneurysm

Computed tomography angiography (CTA) confirmed the presence of a massive, ruptured popliteal artery aneurysm (max diameter 13 cm) (Figure 2).

The patient was driven emergently to the operating room, and under general anesthesia he underwent an open surgical repair (medial approach: superficial femoral-popliteal artery bypass) in a supine position utilizing a ringed PTFE graft (GORE PROPATEN® graft). The postoperative course was complicated by cardiopulmonary arrest on the sixth postoperative day, ultimately leading to the patient’s death.

## Discussion

Considering epidemiology, PAAs may be bilateral or coexist with abdominal aortic aneurysms (AAAs). Approximately 50% of patients suffering from PAA will develop PAA in the contralateral extremity at some time. Also, 50% of individuals with PAA have also AAA [1]. Conversely, in patients with AAA, ~30% also have PAA. Whereas the major concern regarding aneurysm disease is rupture, in the case of PAAs, rupture is not a common presentation, occurring only in 2-5% of patients [2]. The above are mentioned to highlight the importance of screening and surveillance in patients with risk factors. According to available data, ruptured popliteal artery aneurysms (rPAAs) appertain to larger aneurysms and older patients than unruptured PAAs, and patients usually present hemodynamically stable [3]. It is noteworthy that totally thrombosed PAAs can also rupture [4,5].

Regarding diagnosis, this can be challenging because rPAAs typically present with nonspecific symptoms like edema and pain and may mimic other common conditions like deep venous thrombosis or musculoskeletal injuries [2,3]. Taking a thorough medical history helps to unveil common risk factors such as atherosclerosis in other vascular beds and smoking, or even the presence of an aneurysm in the abdomen, which increases the suspicion of PAA presence. Genetic disorders like Marfan and neurofibrosis or vasculitis may also be present [6-8]. Moreover, clinical examination is crucial in vascular surgery, which can sometimes lead directly to diagnosis. For instance, in patients where there is a pain in the extremity and a palpable mass in the popliteal fossa, the diagnosis is moving away from other causes (e.g., a Baker’s cyst) as it is obvious that the palpable mass indicates the presence of an aneurysm. Last but not least, edema may be exacerbated in rPAAs, and the preliminary diagnosis could be deep venous thrombosis. However, especially in large-diameter rPAAs, one can notice the presence of hematoma in the posterior surface of the limb and divert the diagnosis from deep venous thrombosis to that of bleeding. Following the initial clinical examination, the diagnosis is established with computed tomography angiography (CTA), which is the diagnostic modality of choice in this pathology. Duplex scans or magnetic resonance imaging can certainly identify the presence of an aneurysm, but in cases of rupture or ischemia, they are not preferred because they offer nonconclusive data or take too long to perform [2,3].

Considering treatment strategy, this depends on the time of the presentation and the center of referral. In cases where there is prolonged, irreversible ischemia, amputation is the last resort [6,9]. In cases where the limb is salvageable, one can perform open or endovascular therapy. Open surgical repair (OSR) consists of a bypass using a prosthetic, venous, or composite graft with a medial or posterior approach. The most common approach is the medial approach with the patient in a supine position. With this approach, the surgeon can also harvest the great saphenous vein to be used as a graft and perform a fasciotomy. However, with a posterior approach, one can achieve complete evacuation of the hematoma. In the author's belief, OSR is the therapy of choice. In our case, we proceeded in OSR because of compression-related symptoms and because we had to assess truncal arteries and perform distal thromboembolectomy before performing the bypass. Nevertheless, OSR can be very challenging, especially in high-diameter aneurysms where there is dilatation of veins below the knee as an effect of popliteal vein compression. This increases the risk of intraoperative venous injury and bleeding. In the literature, there were cases where ligation alone was the offered therapy because of the presence of a collateral network, but this represents a blocking minority [2,3]. Endovascular therapy using self-expandable covered stent is an acceptable solution, despite it is related to lower patency rates than OSR [1,3]. Also, this therapy does not withdraw the compression-related symptoms in the adjacent structures. However, in surgical emergencies, one should perform what knows best to offer prompt hemostasis and restoration of blood flow.

## Conclusions

Ruptured popliteal artery aneurysms are a rare clinical entity that presents with a misleading symptomatology, potentially leading to a delayed diagnosis. Physicians, including general practitioners and orthopedics, who may confront this condition in the emergency department should maintain an elevated index of suspicion. Treatment can be open or endovascular according to clinical condition and the surgeon’s preference.
